# Graphene-Based Sensor for the Detection of Cortisol for Stress Level Monitoring and Diagnostics

**DOI:** 10.3390/diagnostics12112593

**Published:** 2022-10-26

**Authors:** Alexei Zubarev, Marina Cuzminschi, Ana-Maria Iordache, Stefan-Marian Iordache, Constantin Rizea, Cristiana E. A. Grigorescu, Carmen Giuglea

**Affiliations:** 1 National Institute for Laser, Plasma and Radiation Physics, 077125 Magurele, Romania; 2 Department of Theoretical Physics, Horia Hulubei National Institute of Physics and Nuclear Engineering, 077125 Magurele, Romania; 3 Faculty of Physics, University of Bucharest, 077125 Magurele, Romania; 4 Optospintronics Department, National Institute for Research and Development for Optoelectronics—INOE 2000, 077125 Magurele, Romania; 5 Cabinet Veterinar Roxy Veterinary Magurele, 077125 Magurele, Romania; 6 Department of Plastic Surgery, University of Medicine and Pharmacy “Carol Davila”, 050474 Bucharest, Romania

**Keywords:** cortisol, electrochemical sensors, graphene-based sensing materials, graphene/pyrrole, cyclic voltammetry

## Abstract

In this work, we study the sensing properties of multi-layer graphene combined with pyrrole in order to elaborate low-cost, high-sensitive material for cortisol detection. Graphene nanoplatelets and pyrrole were dispersed in a solution containing 1M HNO_3_ by using a powerful ultrasound probe for 10 min, then centrifuged for 30 min at 4000 rpm; polymerization was performed by cyclic voltammetry. The graphene–pyrrole composite was tested to ultra-low levels of cortisol in artificial saliva, consistent to the levels excreted in human salivary samples. The composite was further investigated by Raman spectroscopy and we modeled the interaction between the sensitive layer and cortisol using MarvinBeans software. It shows a good sensitivity for salivary values of cortisol cyclic voltammetry being able to detect a level down to 0.5 ng/mL cortisol.

## 1. Introduction

Electrochemical sensors are attractive devices with potential applications in quality control of food, water and pharmaceutical compounds [1,2,3]. In recent years, there has taken place an ongoing search in the scientific community for low-cost and high-precision electrochemical sensors [4,5,6]. In general, in the electrochemical sensors the role of the detector is played by the modified electrode in the presence of the investigated substance. High-sensitivity sensors can be produced using carbon-based materials owing to their outstanding conductivity, like for instance, graphene, carbon nanotubes and graphene quantum dots [7,8,9]. Metallic nanomaterials are also good candidates for electrochemical sensors. Some examples include iron oxide nanoparticles, which can be used in detection of analytes like glucose [10] and heavy metals [11]; manganese oxide—detection of hydrogen peroxide [12]; titanium dioxide nanoparticles—detection of p-nonylphenol [13]; copper oxide—detection of non-enzymatic pesticides [14]; and zinc oxide—detection of glucose [15].

Furthermore, electrochemical sensors play an important role in clinical diagnostics. Detection of neurotransmitters is of great importance in investigation of neuro-degenerative diseases because neurotransmitters act as messengers in synaptic transmission. For instance, Wang et al. [16] developed a sensor consisting of flower-like, NiAl-layered double hydroxides and carbon dots for non-enzymatic acetylcholine detection. Acetylcholine is the neurotransmitter responsible for memory, emotions, learning and movement [17]. Glutamate is another neurotransmitter that intervenes in learning and memory processes, and which can be distinguished using a carbon nanofibers and tetrahedral amorphous carbon-based sensor [18]. Dopamine is a neurotransmitter associated with neuronal plasticity, cognition, sleep, movement and learning processes [19]. In Ref. [20], it was shown that electrochemical sensors implemented using gold nanoparticles can be used for detection of dopamine.

Modification of serotonin levels can induce anxiety, eating disorders, sleep disorders and depression. It even can lead to infantile autism. Serotonin is a neurotransmitter that influences muscle contraction and endocrine system regulation [17,21]. Serotonin can be distinguished using a sensor with a glassy carbon electrode covered with carbon nanotubes [21].

Stress is linked to a number of diseases and health issues, such as heart diseases [22], headaches [23], diabetes mellitus [24], asthma [25], autoimmune diseases [26], cancer [27] and more. Cortisol secretion is associated with stress response or low blood–glucose concentration [28]. Cortisol is one of the steroid hormones. Its secretion is implemented by the hypothalamus, pituitary and adrenal glands in the human body.

Cortisol, which is considered a stress hormone, influences the circadian cycle and immune system response [29]. It also regulates blood sugar [30] and can be considered a biomarker for weight gain and obesity risk [31]. Moreover, cortisol hormone governs numerous physiological processes and takes part in homeostasis of the cardiovascular, renal, skeletal and endocrine systems and adjusts blood pressure [32]. Cortisol can be detected in several biofluids, including sweat and saliva, in considerable amounts. Compared to other biological fluids, these two biofluids are the easiest and least invasive to collect.

Carbon-based materials are excellent for cortisol detection. An electrochemical sensor based on screen-printed, multi-walled carbon nanotubes-modified electrodes has been implemented for the detection of cortisol in saliva [33]. The authors have chosen saliva as the most suitable biofluid because of the abundance of the medical information it contains, and non-invasive methods of its collection. The study proved that the device is low-cost and comparable with commercial counterparts. A highly sensitive, carbon fiber-based flexible electrochemical sensor has been implemented and investigated in the study [32]. The sensor exhibits 120 s response time, making it very attractive for the market. Acrylamide polymers, and a fullerene-modified, carbon electrode-based molecularly cortisol imprinted sensor was investigated in study [34]. It was shown that the sensor can detect the cortisol levels in saliva. Another study [35] suggests that graphene-based sensors can be used for cortisol monitorization.

Sensors that use biogenic amines as analytes have found applications in freshness control of meat, seafood and generally, in foods rich in proteins. Biogenic amines are produced by the microbial decarboxylation of amino acids of foods and beverages that have undergone the fermentation process [36]. Platinum electrode with copper plating has been successfully used as an electrochemical sensor for polyamines detection [37]. Tryptamine (a biogenic amine that also plays a role in coordination of biogenic amine-based synaptic physiology [38]) can be detected using a gold nanoparticles and multiwalled carbon nanotube–chitosan-based sensor [39]. In Ref. [40], hydrothermal Ni–MOF (metal-organic framework) was employed to derive a nickel-based sensing agent for histamine.

Due to its exceptional physical and electrochemical properties, graphene is an excellent candidate for electrochemical sensors [8]. Food spoilage that arose as a result of biogenic amines or microbes is a crucial problem in day-to-day life. In Ref. [41], the authors have developed an in-situ receptor-conjugated graphene electronic nose. It allows real-time, high precision monitoring. Sometimes, for better results, graphene can be used together with other materials for electrochemical sensing. For instance, polystyrene–graphene oxide was used as modified glassy carbon electrode for the detection of histamine [42]. Graphene can also be used to detect neurotransmitters. Grass-like laser-scribed graphene has found applications in electrochemical sensing of dopamine, epinephrine and norepinephrine [43]. Adjusting the laser-scribing parameters it has obtained a unique morphology of graphene with peculiar properties, useful in the context of electrochemical sensors.

To the best of our knowledge, there are few attempts to use a graphene–polypyrrole composite to detect cortisol in a synthetic saliva matrix. Manickam et al. in 2017 used a molecularly imprinted polymer technique for the development of a polypyrrole-based sensor for cortisol in saliva [44], while Borazjani et al. used a graphene–polypyrrole architecture for discrimination of mandelic acid enantiomers in the presence of hydrocortisone as chiral selector [45]. Building on their experience, we carried out the synthesis of the composite material in a simpler manner, following a “green” approach. To improve the theoretical studies over the recognition mechanism, we employed a modeling software which could allow a simulation of the reaction between graphene–polypyrrole and hydrocortisone. In this work we propose to elaborate a novel, high-sensitive sensor based on multilayer graphene and pyrrole for cortisol detection in saliva. We choose graphene and pyrrole due to their eco-friendly synthesis [46,47,48], their safety towards human application [49,50] and because of the low-cost method. In the following sections, we describe the materials and fabrication of the graphene–pyrrole compound, our results after electrochemical characterization of the compound in the presence of cortisol, we calculate its linearity of the current density for the reduction peak of hydrocortisone over the concentration range and we modeled the interaction between cortisol and composite using MarvinBean® software.

## 2. Materials and Methods

### 2.1. Reagents and Equipment

Carboxymethylcellulose sodium salt (CMC 98%, Sigma Aldrich), potassium chloride (99%, Sigma Aldrich), calcium acetate hydrate (99%, Alfa Aesar), potassium phosphate monobasic (99%, Alfa Aesar), dipotassium phosphate (99%, Alfa Aesar), pyrrole (99%, Alfa Aesar) and all other chemical compounds were used as delivered, without purification. The graphene nanoplatelets were purchased from Sigma Aldrich. We could not procure human cortisol, so we purchased the drug hidrocortizon hemisuccinat (Zentiva) from a local pharmacy. C11L sensor support (Metrohm DropSens S.L., Llanera, Spain) have working and auxiliary electrodes made of carbon and the reference electrode made from Ag/AgCl. They were used as support for the electropolymerization of the graphene–pyrrole composite.

Electrochemical characterization was performed with OrigaFlex 0.5A modular electrochemical system from OrigaLys ElectroChem SAS (Rillieux-la-Pape, France) and a three-electrode cell. Raman spectroscopy was performed with a LABRAM HR 800 spectrometer with the excitation laser wavelength 633 nm and laser power at the sample surface of 2.11 mW for each measurement. MarvinBeans^®^ (Chemaxon, Basel, Switzerland) was employed to model the reaction of cortisol on the composite. The modelling of the reaction was based on the assumptions that pyrrole is polymerized on the edges of the graphene sheet and that hydrocortisone will react with the nitrogen atoms of the polymer via carboxyl/hydroxyl groups.

### 2.2. Composite Solution Preparation

A quantity of ≈ 1.0 mg graphene nanoplatelets and 600 µL pyrrole were dispersed in 50 mL 1M HNO_3_ with a Hielscher ultrasound probe (Hielscher Ultrasonics, Teltow, Germany) and centrifuged at 4000 rpm for 30 min to separate the deposit from the supernatant. The supernatant was collected and used in the following electrochemical polymerization. The amounts of precursor were selected under the principle of “green chemistry”—atom economy, which requires only the minimal amount of reactants to produce the composite (lowering the quantities is time consuming and requires more energy to produce the same result, while increasing the amount will be a waste of precursors).

### 2.3. Electrochemical Polymerization of Graphene–Pyrrole Composite

The C11L electrode surface was washed and dried with an IR lamp before polymerization. As a mask for the auxiliary and reference electrodes, we used parafilm which was wrapped around the sensor keeping only the working electrode free and in contact with the composite solution. The supernatant was placed in the electrochemical cell, together with the saturated reference calomel electrode and a Pt wire acting as the auxiliary electrode. The potential was swept between 2,212,800 MV and 800 mV, for 20 cycles, with a scan rate of 100 mV/s. After polymerization, the sensors were washed in deionized water, and dried again with an IR lamp before use.

### 2.4. Preparation of the Artificial Saliva

We prepared artificial saliva samples based on the protocol of Macknight-Hane and Whitford, in 1992 [51]. Sorbitol and methyl-p-hydroxybenzoate were omitted because we needed a more fluid and “greener” solution. Thus, our formulation for the artificial saliva became: 2.5 g sodium carboxymethyl cellulose (CMC) dissolved in 50 mL boiling distilled water, 0.159 g KCl, 0.02 g Ca acetate hydrate, 0.19 g K_2_HPO_4_ and 0.08 KH_2_PO_4_. This solution was, however, too thick. Therefore, we diluted it with 230 mL distilled water. After two hours on the heating plate and under magnetic stirring, the solution had the consistency of a running saliva. The pH of the artificial saliva was 7, at 24.2 °C.

### 2.5. Preparation of the Cortisol Solutions

We wanted our solution to match the biological reference interval in salivary samples for cortisol, which is 0.5–5 ng/mL. Thus, we prepared a stock solution of 100 ng/mL hydrocortisone in artificial saliva and by successive dilutions we prepared six samples with concentrations in the biological intervals: 0.5 ng/mL, 1 ng/mL, 2 ng/mL, 3 ng/mL, 4 ng/mL and 5 ng/mL. 50 µL of each solution was dropcasted onto the modified electrode and tested via cyclic voltammetry.

## 3. Results and Discussions

### 3.1. Electropolymerization of Graphene–Pyrrole

**Figure 1:**
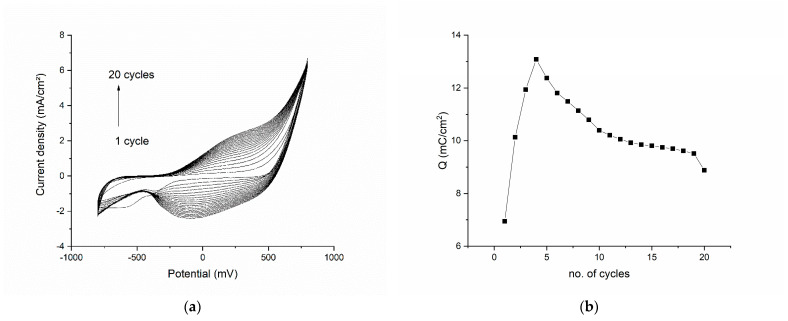
Electropolymerization results for the graphene–pyrrole composite: (**a**) the voltammogram of each of the 20 cycles of the polymerization procedure; (**b**) the total charge exchanged in the polymerization process as function of the number of cycles.

The results of the electropolymerization cycles are presented in Figure 1. The voltammogram shows each of the 20 cycles from the polymerization procedure (Figure 1a). We can see that the first cycle has a broad reduction peak at −550 mV, corresponding to the reduction of an element in the 1M HNO_3_ electrolyte solution or on the surface of the modified electrode (an impurity). In the subsequent cycles, this reduction peak disappears and is replaced by another broad reduction plateau in the range −100 to −200 mV. The oxidation peak is also broad and begins to be well defined after cycle 4. The shape of the reduction and oxidation peaks can be explained by the poor conductivity of the pyrrole in the beginning of the polymerization, conductivity which enhances as the number of cycles increases. By plotting the total charge exchanged in the polymerization process vs. the number of cycles (Figure 1b) we can clearly see that the charge increases drastically for the first four cycles, only to begin to drop in all the subsequent cycles. This confirms the deposition of a polymeric film onto the working electrode. Another important observation is that the charge decreases slowly after cycle 4, which suggests that the polymeric film is conductive (due, in part, to the graphene as well as to polypyrrole) and the diffusion process is slightly impaired by the composite film.

The double-charged layer in the polymerization graphs increases as the number of layers on the surface of the electrode increase, but access to the working electrode surface is maintained through pores. This is evident since the oxidation and reduction waves at 500 mV are constant throughout the CV.

### 3.2. Electrochemical Investigation of the Graphene–Pyrrole Composite in Artificial Saliva

**Figure 2:**
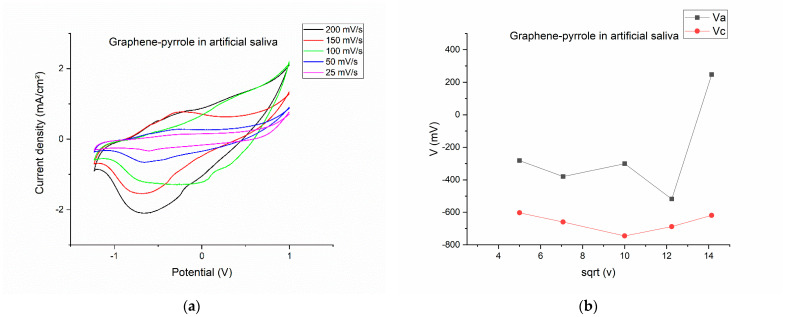

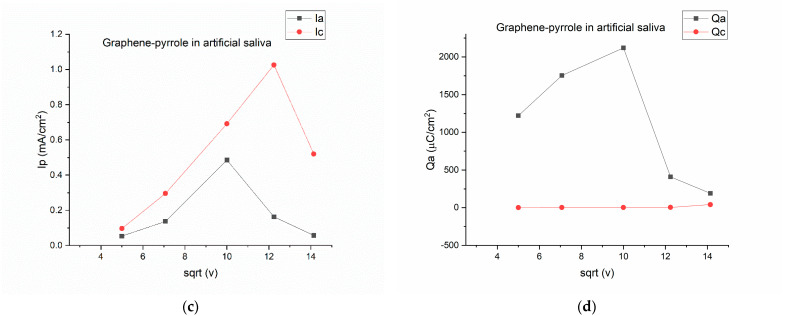
Electrochemical behavior of the composite in artificial saliva: (**a**) variation of the current-voltage characteristics at different scanning rates; (**b**) anodic/cathodic potential as function of the square root of the scanning rate; (**c**) anodic/cathodic peak intensity as function of the square root of the scanning rate; (**d**) total charge vs. the square root of the scanning speed.

After polymerization, the modified electrode was washed, dried and tested to the artificial saliva solution. Figure 2 shows the results of this test. The CVs were recorded at different scanning rates (Figure 2a) indicating that at high scanning rates the signal becomes distorted (at 100 mV/s, the potential of the reduction and oxidation peaks are shifted completely differently than all the other scanning rates). This becomes interesting because for 150 mV/s, the potential for both oxidation and reduction is proportional with the ones seen at lower speeds. At 200 mV/s, the oxidation peak is completely diminished, but the reduction peak is the most prominent. At lower speeds, we can see that the oxidation and reduction peaks produce a reversible response on the CV.

By plotting the parameters of the voltamogram vs. the square root of the scanning speed, we can see the complexity of the reactions on the surface of the electrode. On Figure 2b, the potential is represented vs. the square root of the scanning rate and is obvious that for the 200 mV/s speed the potential for the oxidation is drastically shifted to a positive value, considering that all the other potentials have a negative value. The most stable is the reduction peak, which has a close-to-monotone variation. The same thing is observed in Figure 2d, where the charge for the cathodic peak is monotone, whereas the charge exchanged during oxidation has a non-linear response. This means that the artificial saliva actively interacts with the composite on the electrode, but a pinpoint of the reaction elements is difficult due to the multitude of chemical species present in the solution. Figure 2c gave us hope that the reduction peak should be the one to be pursued if a quantification of the cortisol is to be made.

### 3.3. Detection of Cortisol in Artificial Saliva

**Figure 3:**
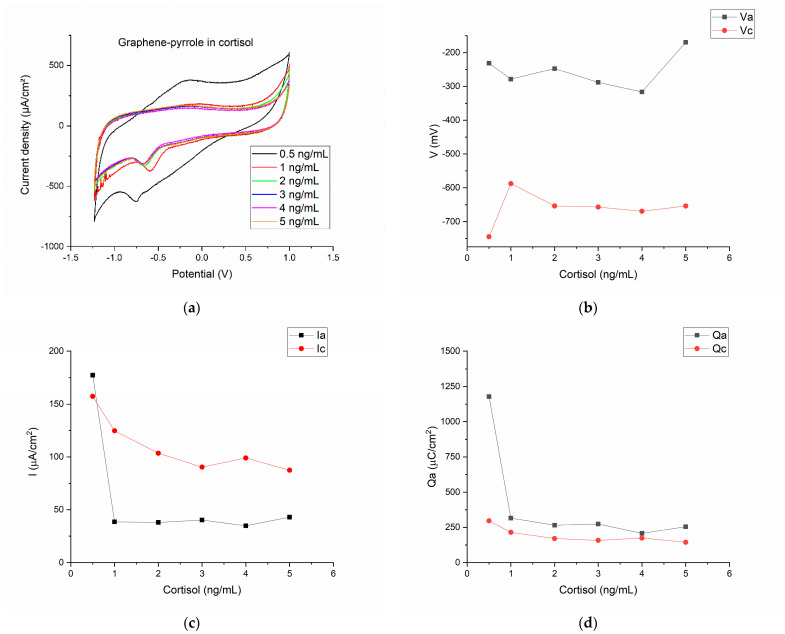
Electrochemical results for the detection of cortisol in artificial saliva with the composite material: (**a**) the cyclic voltammograms for the six concentrations of hydrocortisone in artificial saliva; (**b**) the variation of the anodic/cathodic potential with the concentration of the cortisol samples; (**c**) anodic/cathodic peak intensity variation as function of the cortisol concentration; (**d**) the charge transfer for the anodic/cathodic processes as function of the different cortisol concentrations.

Figure 3a shows the cyclic voltammograms for the six concentrations of hydrocortisone in artificial saliva. The first observation is that now the reduction peak is well defined and at a more negative potential than the reduction plateau. Figure 3b shows the variation of the potential vs. the concentration of the cortisol samples. The two peaks are clearly separated with the oxidation peak in the interval (−200–−300 mV) and the reduction peak positioned at even more negative potentials (−600–−700 mV).

The current density associated with the reduction and oxidation peaks shows that only for the lowest concentration of cortisol, the current density of the anodic wave is superior to the reduction peak. Actually, for the 0.5 ng/mL cortisol, the ratio Ipa/Ipc is close to the unity (Ipa/Ipc = 1.12). For all the other concentrations, the current density ratio is ranging from 0.3–0.49 (this is seen in the Figure 1c, as the cathodic current has greater values than the anodic current). However, the charge transfer is relatively similar for the oxidation and reduction for the six cortisol concentrations (Figure 3d).

Figure 3 indicates that cortisol can be actively monitored by the composite, but the response of the sensor is not monotone and does not perfectly fit a linear equation. In fact, the equation we obtained by linear fitting the cathodic peak intensity was:

(1) $$I_{pc}=143.79532-12.91974\cdot x$$

with R^2^ = 0.72187.

This value means that only 70% of the cases will fall in the correct interval and the rest will be false values. The correlation factor is lower than expected (from the reduction peak seen in the CVs, we expected a correlation factor ˃ 0.85) but it can be explained by the low concentrations of cortisol in saliva. Compared to blood concentrations, salivary cortisol is at the ng/mL level (almost trace level). Previous studies concluded that the connection between cortisol and mental load depends on the individual; cortisol secretion is dependent on what the individual think is stressful (e.g., sitting on a chair can be perceived by some people as comfortable [52]).

### 3.4. Raman Spectroscopy

**Figure 4:**
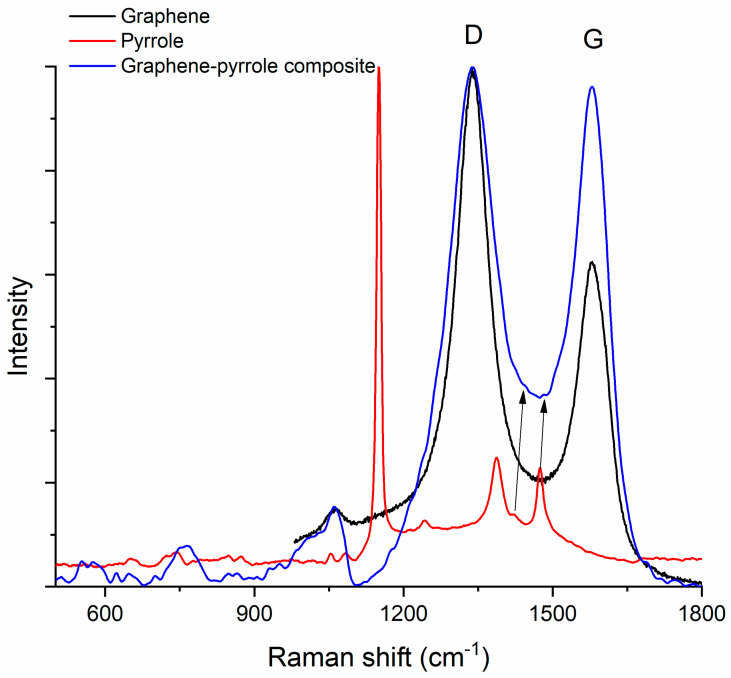
Raman spectrum of the composite compared to the Raman spectra of the constituents. The arrows indicate the low intensity peaks at 1386 and 1476 cm^−1^ associated with pyrrole.

Figure 4 shows the Raman spectra of pyrrole, graphene and the composite. The graphene spectrum presents the two characteristic bands D (1338 cm^−1^) and G (1579 cm^−1^) [53], while the pyrrole spectrum shows the C–H in plane deformation of oxidized polypyrrole at 1151 cm^−1^, the inter-ring stretching of the C–C bond at 1386 cm^−1^, the C–C and C–N stretching at 1476 cm^−1^ and antisymmetrical in-plane bending of the C–H bond at 1241 cm^−1^ [54]. In the composite material, the D-band is conserved and observed at 1338 cm^−1^ but the peak is “broader” which signifies that the dimensions of the graphene particle have increased. The G-band increases also due to polymerization of the pyrrole and bonding of the pyrrole to the graphene sheet. The peaks for the graphene are better represented in the composite, while the peaks for the polymer appear as shoulders (this is observed at the base of the major peak of the composite, where we can almost “see” the peaks at 1386 and 1476 cm^−1^ associated with pyrrole). The position of the G-band in the spectrum of the composite remains the same as in the graphene spectrum but the ratio I_D_/I_G_ is modified: from 1.86 in graphene it drops to 1.06 in the composite material. This decrease of the I_D_/I_G_ ratio indicates an increase of the disorder (according to Ferrari [55], the D-band appears at 1332 cm^−1^ in highly ordered carbons, e.g., diamond, and is produced by sp^3^ atoms; but for all other carbon materials, the D-band corresponds to the breathing modes of sp^2^ atoms in rings). We assume this happens because the pyrrole is attached to the margin of the graphene rather than on top of the sheet [56,57].

### 3.5. MarvinBean^®^ Interaction Model

**Figure 5:**
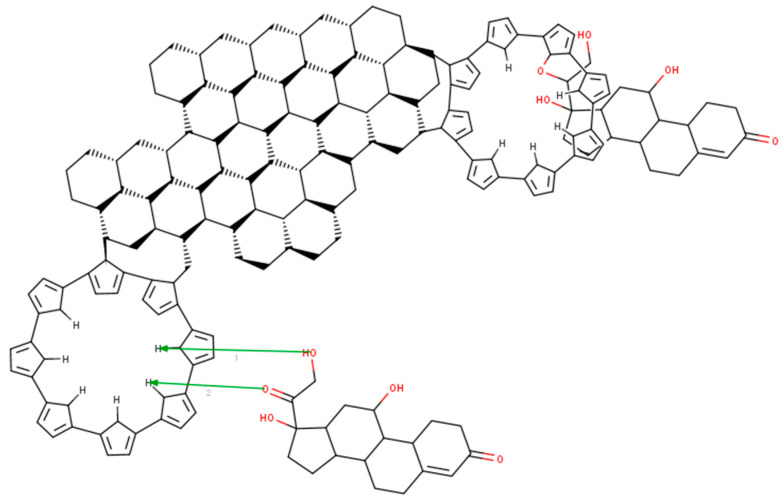
Modeling of the reaction between the graphene–pyrrole composite and cortisol with MarvinBean^®^.

In order to better explain the way the composite and hydrocortisone interact, we developed a model for the composite–cortisol reaction. Even if the model in Figure 5 is 2D, we can easily see that the polypyrrole will attach itself onto the edge of the graphene plate and will form “a plate”. Hydrocortisone will snuggle inside the plate by bonding to the nitrogen atom in the center of the polymer via carboxyl/hydroxyl groups. During this reaction, water will be released in the electrolyte. However, hydrocortisone can bond to the polypyrrole molecule on the out-side plane too. In fact, polypyrrole acts as antennae for the hydrocortisone. Another aspect is that the polypyrrole forms “crowns”, which increase the porosity of the composite (this is sustained by the electrochemistry, because there too we found that the composite was porous and allowed free flow to and from the surface of the electrode).

Perspectives: The following steps in the development of the cortisol sensor are: (a) Interferents measurements—we need to analyze the way other molecules common in human saliva could interfere with cortisol detection. This is particularly important when considering the salivary microbiome on top of other common interferents (e.g., glucose). (b) Reproducibility—we need to synthesize a larger number of sensors and to test all of them in the same conditions in order to establish the reproducibility of the synthesis method. (c) Another important aspect is the stability of the sensor in different conditions (considering that this sensor is aimed at assessing the cortisol in saliva, we exclude strong acids and bases, but we need to test it to ˃ 160 mM HCl which is the concentration of stomach acid). (d) Miniaturization and incorporation into a wearable device similar to a holter monitor—this involves processing materials and integration into microfluidic channels.

## 4. Conclusions

In summary, a graphene and pyrrole-based sensor has been investigated in this paper for detection of cortisol hormone levels in artificial saliva. The selected materials, graphene and pyrrole, allow for green chemistry implementation and exhibit the most needed properties such as safety and low cost. Graphene and pyrrole nanostructures were diffused in solution with 1M HNO_3_ via ultrasound, after that centrifugation was implemented and cyclic voltammetry was acquired for polymerization. The composite was tested for six different ultra-low concentrations of cortisol in artificial saliva: 0.5 ng/mL, 1 ng/mL, 2 ng/mL, 3 ng/mL, 4 ng/mL and 5 ng/mL. We studied the spectroscopic properties of the composite via Raman spectroscopy. Moreover, we used MarvinBeans® software to visualize the interaction between cortisol and the graphene–pyrrole composite; the developed model was in accordance with the results obtained by electrochemical experimentation and provided a “best-guess” snapshot of the reaction.

## Data Availability

Data are available upon request to the corresponding authors in reasonable time frame.
